# Comprehensive Analysis of Splicing Factor and Alternative Splicing Event to Construct Subtype-Specific Prognosis-Predicting Models for Breast Cancer

**DOI:** 10.3389/fgene.2021.736423

**Published:** 2021-09-24

**Authors:** He Zhang, Baoai Han, Xingxing Han, Yuying Zhu, Hui Liu, Zhiyong Wang, Yanfen Cui, Ran Tian, Zicong Gao, Ruinan Tian, Sixin Ren, Xiaoyan Zuo, Jianfei Tian, Fei Zhang, Ruifang Niu

**Affiliations:** ^1^ Public Laboratory, National Clinical Research Center for Cancer, Tianjin Medical University Cancer Institute and Hospital, Tianjin, China; ^2^ Key Laboratory of Cancer Prevention and Therapy, Tianjin, China; ^3^ Tianjin’s Clinical Research Center for Cancer, Tianjin, China; ^4^ Key Laboratory of Breast Cancer Prevention and Therapy, Ministry of Education, Tianjin, China

**Keywords:** breast cancer, TCGA database, prognosis, splicing factor, alternative splicing

## Abstract

Recent evidence suggests that splicing factors (SFs) and alternative splicing (AS) play important roles in cancer progression. We constructed four SF-risk-models using 12 survival-related SFs. In Luminal-A, Luminal-B, Her-2, and Basal-Like BRCA, SF-risk-models for three genes (*PAXBP1*, *NKAP*, and *NCBP2*), four genes (*RBM15B*, *PNN*, *ACIN1*, and *SRSF8*), three genes (*LSM3*, *SNRNP200*, and *SNU13*), and three genes (*SRPK3*, *PUF60*, and *PNN*) were constructed. These models have a promising prognosis-predicting power. The co-expression and protein-protein interaction analysis suggest that the 12 SFs are highly functional-connected. Pathway analysis and gene set enrichment analysis suggests that the functional role of the selected 12 SFs is highly context-dependent among different BRCA subtypes. We further constructed four AS-risk-models with good prognosis predicting ability in four BRCA subtypes by integrating the four SF-risk-models and 21 survival-related AS-events. This study proposed that SFs and ASs were potential multidimensional biomarkers for the diagnosis, prognosis, and treatment of BRCA.

## Introduction

Alternative splicing (AS) is a post-transcriptional process, in which the precursor mRNA is processed to different mature mRNAs from one protein-coding gene (Baralle and Giudice, 2017). The AS is a highly regulated process and nearly all multiexon genes are involved in AS and most cellular processes may be regulated by AS (Cherry and Lynch, 2020; Sciarrillo et al., 2020). In this process, genes can be edited into many different mature mRNAs to produce different proteins. AS includes seven types of alternative splicing, namely, alternate acceptor site (AA), alternate donor site (AD), alternate promoter (AP), alternate terminator (AT), exon skip (ES), mutually exclusive exons (ME), and retained intron (RI) (Taylor and Sobczak, 2020). AS modifies more than 90% of human genes and is an important mechanism to enhance transcription and protein diversity by including or excluding different exons or partial exons in mRNA (Raj and Blencowe, 2015; Baralle and Giudice, 2017; Martinez-Montiel et al., 2018). Therefore, AS events are closely related to many physiological and pathological processes including cancer. However, the clinical relevance of ASs has largely remained unexplored.

The disorder of AS can promote the occurrence of tumors and affect the key phenotypes of tumor cells, such as proliferation, apoptosis, invasion, and metastasis (Oltean and Bates, 2014). Emerging evidence suggests that tumor initiation and progression are complex processes that cannot simply be attributed to the misregulation of “tumor suppressor” or/and “oncogenes” (Hjelmeland and Zhang, 2016). Alternative splicing could be one of the important reasons for this “complex” situation. Some isoforms of “oncogenes” can promote the apoptosis of tumor cells, but some “tumor suppressor genes” can enhance the migration and invasion ability of tumor cells after deleting an important domain (Sveen et al., 2016). Several genes have been reported to be involved in alternative splicing, and the splicing isoforms may serve as potential biomarkers and therapeutic targets (Zhang et al., 2019a). One of the most extensively studied genes is the apoptosis-related gene *BCL-X* which encodes two protein isomers, namely, BCL-XL and BCL-XS, these isomers have opposite effects on apoptosis (Shiraiwa et al., 1996; Bauman et al., 2010). Other examples also emerge, such as the splicing isoforms of *AIMP2*. *BRCA5*, *BRAF*, *VEGFA* and *CXCL12* are associated with lung cancer, ovarian cancer, melanoma, colorectal cancer, and breast cancer (BRCA), respectively (Choi et al., 2011; Poulikakos et al., 2011; Vivas-Mejia et al., 2011; Bates et al., 2012; Zhao et al., 2014). The dysregulation of the splicing factor (SF) expression may lead to overall changes in some cancer-specific AS events, thereby affecting the occurrence and development of cancer (Urbanski et al., 2018). The proportion of BCL-XL and BCL-XS is regulated by some SFs, such as SAM68, which can promote the expression of BCL-XS and induce apoptosis (Paronetto et al., 2007; Bielli et al., 2014). These findings indicate that the potential splicing factor-alternative splicing (SF-AS) regulatory network provides a new perspective for exploring tumor biomarkers and tumorigenesis mechanisms.

BRCA is one of the most common malignant tumors in women, accounting for 30% of all newly diagnosed cancers, and the second leading cause of cancer death in women worldwide (Li et al., 2016; Ahmad, 2019). Although advanced diagnosis and therapeutic strategies have significantly extended the survival-time of BRCA patients, BRCA still remains a major life-threatening factor among women worldwide. BRCA can be classified into four different molecular subtypes, namely, Luminal-A, Luminal-B, Her-2, and Basal-like, known as PAM50 intrinsic molecular subtype (Nielsen et al., 2010). The PAM50 subtypes can be determined by immunohistochemistry (IHC). Luminal A features ER/or PR positive IHC signatures, Luminal B approximates ER and/or PR positive plus HER2 positive signatures, HER2 features ER and PR negative, but its HER2 is strongly positive. However, Basal-like BRCA has all ER, PR and HER2 negative under IHC examination (Hon et al., 2016; Sun et al., 2019a).

Biological properties and molecular characteristics are different among the four types of BRCA. The therapeutic strategies, as well as the prognosis, are also highly dependent on the molecular subtype of BRCA patients (Inoue and Fry, 2015; Wallden et al., 2015; Cao and Niu, 2020). The distinct molecular characteristics between different molecular subtypes are also interconnected with AS events to form a unique molecular environment in different BRCA subtypes, thereby modulating the biological properties of cancer cells (Climente-González et al., 2017). Studies have been conducted extensively to demonstrate the role of AS events on BRCA. Results have shown that abnormal splicing of ER and HER2 correlated to the occurrence of BRCA; thus, it may be a potential target for cancer treatment (Inoue and Fry, 2015; Martínez-Pérez et al., 2019; Francies et al., 2020). Dysregulation of SFs has also been investigated, and results have shown that they are connected to the malignant phenotype of BRCA (Park et al., 2019). Thus, SFs and ASs potentially serve as diagnostic markers to predict patients’ clinical outcomes. However, few studies have provided a genome-wide landscape of SF and AS in four BRCA subtypes, and the clinical significance of these SFs and ASs is poorly described.

In the present study, we comprehensively analyzed the expression patterns of SFs and ASs in BRCA with different molecular subtypes. Furthermore, four subtype-specific SF risk models were constructed using the survival-related SFs. The potential mechanistic connections in the risk models were described using a series of bioinformatics approaches. Lastly, we identified key AS events and constructed four subtype-specific AS risk models by using a combination of SF risk models and survival-related AS events. The constructed AS/SF risk models may provide new multidimensional biomarkers for the prognosis and diagnosis of BRCA with different molecular subtypes.

## Materials and Methods

### Data Collection and Preprocessing

The RNAseq data were downloaded from *The Cancer Genome Atlas (TCGA)* database and transformed to transcripts per million (TPM) for downstream analysis (https://portal.gdc.cancer.gov/). The batch effect in the data was analyzed by *TCGA Batch Effects Viewer* (https://bioinformatics.mdanderson.org/public-software/) and no significant batch effect was found. The clinicopathological information was obtained using *Xnea* database (http://xena.ucsc.edu/). The detailed clinicopathological information of BRCA patients involved in this study is described in Supplementary Table S1. The gene list of SFs was obtained from a previous pan-cancer study conducted by Seiler et al. (2018) and 393 SFs (Supplementary Table S1) were subjected to downstream analysis after omitting unmatched gene symbols.

The RNA splicing data were downloaded from *TCGA SplicingSeq* database (https://bioinformatics.mdanderson.org/TCGASpliceSeq/index.jsp). The percent spliced index (PSI), an intuitive ratio to quantifying splicing events from 0 to 1, was calculated for seven types of AS patterns: mutually exclusive exons (ME), exon skip (ES), retained intron (RI), alternate terminator (AT), alternate promoter (AP), alternate acceptor site (AA), alternate donor site (AD). AS-events with >30% of “NA” value were omitted to form the study and then the data were processed by the *Impute* package. Then, the imputed AS data were filtered by the standard deviation (SD), and AS-events with an SD < 0.15 were excluded from the study (the preprocessed data contains 910 BRCA samples and 1048575 AS-events). The mRNA expression data were log2 transformed and the PSI data were Z-normalized and then subjected to the downstream analysis. The clinical data were manually curated and cases with incomplete survival data were omitted from the downstream analysis.

### Analysis of the Expression and Survival Landscapes of SFs and ASs

The expression of SFs and ASs were analyzed using principal component analysis (PCA). For mRNA expression data, the expression value of SFs was extracted and log2 transformed. The PSI value of AS data was directly subjected to the PCA. The analysis was done using *pca3d* package in R. For survival analysis, the processed TPM and PSI data were subjected to univariable Cox analysis using *survival* package in R. The survival-related AS-events were also processed by *UpsetR* package to generate the UpSet graph, which is a visual technique for quantitative analysis of interactive sets.

### Construction and Validation of the Subtype-Specific Risk SF-Risk-Models

For the construction of SF-risk-models, the patients (Luminal A, *N* = 528; Luminal B, *N* = 203; HER2, *N* = 76; Basal-like, *N* = 180) were randomly divided into the training group and the testing group, both of them consist 50% of the involved cases. Initially, univariable Cox-analysis was performed to identify the survival-related SFs. Then, the LASSO regression (using glmnet R package) was performed to eliminate the false-positive parameters caused by overfitting. The lambda selected in the regression was determined by “*cvfit$lambda.min*” in “*glmnet*” R package and showed in Supplementary Figure S2A. Finally, the multivariable-Cox analysis was used to calculate the Hazard-Ratio and generated the prognosis model. The RiskScores (SF-RiskScore) for SF-risk-models were calculated by the regression coefficient of a single gene and the expression value of each gene. The LASSO-COX regression was run in a repeated loop for selecting the best gene combination to construct the final prognosis models. So gene combinations with a *p* < 0.05 of Kaplan-miller analysis in both testing and training dataset and ROC-AUC >0.65 were selected.

The calculation formula is as follows:

$$\mathrm{SFRiskScore}=\sum_{i=1,2,3,i}coefficient\;\left(SFi\;\right)\times expression\left(SFi\right)$$

where SFi represents the identifier of the *i*th selected SF. The value of coefficient (SFi) is the regression coefficient estimated by SFi based on Cox proportional risk regression analysis. The RiskScore is a measurement of the prognostic risk of each BRCA patient. The median of RiskScores was used to stratified patients into subgroups.

The performance of the SF-risk-models was further analyzed by Kaplan-Meier analysis and Time-dependent ROC analysis, whereas a log-rank *p* value < 0.05 and area under curve (AUC) > 0.700 were considered as models with acceptable predicting power.

### Construction the Subtype-Specific AS-Risk-Models

For the construction of the subtype-specific AS-risk-models, the AS-events PSI data were firstly Z-score normalized across all the BRCA samples. Then the univariable-Cox analysis was performed to analyze the survival-related AS-events, whereas *p* < 0.05 was considered as statistically significant. Besides, the *Pearson’s* correlation coefficients were calculated using the SF-RiskScore (RiskScore calculated by SF-risk-models) and the PSI value, AS-events with |R| > 0.15 and *p* < 0.05 were screened out. Then we pick the AS-events that both significant in Cox analysis and correlation analysis. The selected AS-events were then subjected to LASSO regression analysis and multivariable-Cox analysis to generate the final prognosis model. The RiskScore (AS-Riskscore) was calculated as follow:

$$\mathrm{ASRiskscore}=\sum_{i=1,2,3,i}coefficient\;\left(ASi\;\right)\times PSI\left(ASi\right)$$

where ASi represents the identifier of the *i*th selected AS. The value of coefficient is the regression coefficient estimated by ASi based on Cox proportional risk regression analysis. The risk score is a measurement of the prognostic risk of each BRCA patient. The median of RiskScore was used to stratified patients into subgroups. The AS-risk-models were evaluated by Kaplan-Meier and Time-dependent ROC analysis. Models with a log-rank *p* value <0.05 and AUC >0.700 were considered have acceptable predicting power.

### Comprehensive Analysis of SFs in the Risk-Specific Model

The SFs involved in the SF-risk-models were subjected to several downstream analyses. The Pearson’s correlation coefficients were calculated between all the 12 SFs and the result was plotted using *corrplot* R package.

The clustered heatmap was plotted using the expression value of 12 SFs and the clinical information for all BRCA patients. Expression of SFs in different molecular subtypes of BRCA was presented using violin plots, the *Wilcox* test and *Kruskal-walls* tests were used to determine the statically significant differentially expressed genes. The SFs in AS-risk-models were also subjected to Kaplen-Meier analysis using the *GEPIA* database (http://gepia.cancer-pku.cn/), the log-rank test was used to determine the statistical significance. The clinical relevance of SFs in Luminal A and Luminal B AS-risk-models were also validated by *Kaplan-Meier plotter* database (http://kmplot.com/), we used progress-free survival time (PFS) to perform the statistical tests.

The 12 SFs were also analyzed by the *cBioProtal* database (http://www.cbioportal.org/) to assess the copy number variation and mRNA expression variation. The threshold to determine the mRNAs expression alteration was set as Z-score = 1.5.

For protein-protein interaction (PPI) network construction, the 12 selected SFs were subjected to *STRING* database (https://string-db.org/), the interacting proteins (both experimentally determined and computational predicted) are marked as colored lines between genes.

Genes involved in the SF-risk-models were also analyzed by *GSCALite* database (http://bioinfo.life.hust.edu.cn/web/GSCALite/) to address the SFs associated tumor-essential pathways.

The mRNA expression of these SFs in tumor and normal tissue were also analyzed by the GEPIA database in four BRCA subtypes, SF with a *P* (Limma method) < 0.01 and |Log2 FC| > 0.5 were regarded as significantly expressed SFs.

### Gene Set Enrichment Analysis

To explore the hallmarks and pathways that were enriched in the predicted high- and low-risk group, Gene set enrichment analysis (GSEA) was performed as previously described (Zhang et al., 2020). Using GSEA, the present study tested whether the activated/repressed gene signatures were enriched for high-risk vs. low-risk cases. The enrichment of pre-defined hallmarks and KEGG pathways was calculated using a normalized enrichment score (NES) and normalized *p*-value. Terms with |NES|>1 and *p* < 0.05 were considered significantly enriched.

### Statistical Analysis

All statistical analyses were performed using R software (version 3.6.0). *p* < 0.05 was considered statistically significant. Wilcox test or *Kruskal–Wallis* test was used to evaluate the distribution differences among variables. *Kaplan–Meier* survival curve analysis and log-rank test were used to analyze OS. The Cox regression model was used to analyze the factors influencing the survival of BRCA patients. Cox proportional risk regression model was used for univariable and multivariate analyses. Time-related ROC analysis was used to assess the accuracy of models for predicting prognosis. We used the survival time, survival state, and RiskScore obtained from the risk models to draw the ROC curve in the R software using the *survivalROC* package, and both 5, 3 and 10 years ROC curve was drawn. The AUC value greater than or equal to 0.70 was regarded as the significant prediction value, and AUC value greater than or equal to 0.65 was regarded as the acceptable predicted value.

## Results

### Flowchart of this Study

**FIGURE 1:**
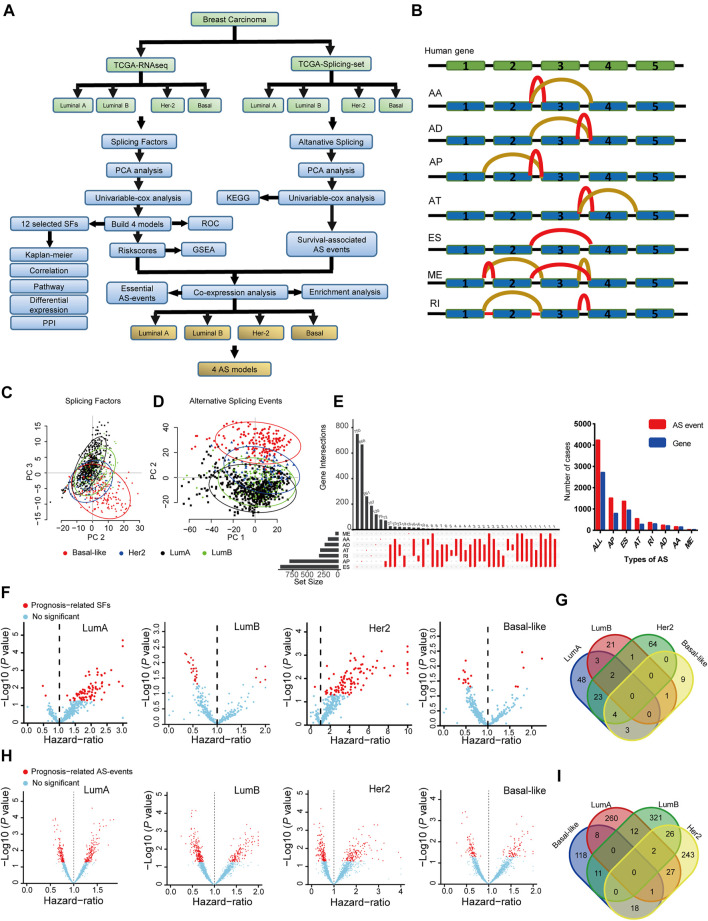
The landscape of splicing-factors and alternative-events in Luminal-A, Luminal-B, Her-2 and Basal-like BRCA. **(A)** The follow chart of this study. AA (alternate acceptor site), an alternative 3′ splice junction (acceptor site) is used, changing the 5′ boundary of the downstream exon. AD (alternate donor site), an alternative 5′ splice junction (donor site) is used, changing the 3′ boundary of the upstream exon. AP (alternate promoter), an alternative region from which transcripts of a gene originate. AT (alternate terminator), the transcripts have more than one termination exons. ES (exon skip), an exon is removed with its intron-flanking site. ME (mutually exclusive exons), exons do not occur together but do not refer to length, sequence or exon numbers. RI (retained intron), an intron retains in the final transcript. The red and brown line indicates different final transcripts resulted from the corresponded alternative splicing event. **(B)** Schematic summary diagram of alternative splicing events analyzed in this study. **(C)** Principal Component Analysis (PCA) of the splicing-factors in Luminal-A, Luminal-B, Her-2 and Basal-like BRCA. **(D)** PCA of splicing-events in Luminal-A, Luminal-B, Her-2 and Basal-like BRCA. **(E)** UpSet plot and bar plot showing the statistics of seven types of AS-events in BRCA. For the same splicing events that happened in different patients, the events were counted only once. **(F)** Volcano plots showing the Hazard-Ratio (HR) and log10-*P* value of splicing-factor in univariable-Cox analysis in four subtypes of BRCA. **(G)** Venn diagram showing the intersections of survival-significance splicing-factors in four subtypes of BRCA. **(H)** Volcano plots showing the Hazard-Ratio (HR) and log10-*P* value of alternative-splicing events in univariable-Cox analysis in four subtypes of BRCA. **(I)** Venn diagram showing the intersections of survival-significance alternative-splicing events in four subtypes of BRCA.

The detailed workflow of this study is shown in Figure 1A. We first download The Cancer Genome Atlas (TCGA)-RNAseq data, TCGA alternative splicing PSI data, and the related clinical data from TCGA Data Portal and TCGA SplicingSeq database. A preprocessing step was implemented to improve the data quality for downstream analysis.

Then, we use PCA and univariable-Cox analysis to address the expression pattern and clinical relevance of all SFs and ASs. The survival-related AS was subjected to KEGG enrichment analysis. Four SF risk models (containing 12 SFs) were constructed using survival-related SFs, and the performance of these models was verified by ROC and Kaplan-Meier analysis in the testing and training datasets. The expression, clinical relevance, and potential mechanical links in BRCA were further described by a combination of Kaplan-Meier analysis, mRNA correlation analysis, cancer-related pathway analysis, mRNA differential expression analysis, and PPI analysis. Lastly, we combine four constructed SF risk models and survival-related AS events to screen essential AS events, and four AS risk models were built with high prognosis predicting capacity. The schematic representation of analyzed AS events is shown in Figure 1B.

### The Landscape of SFs and Alternate Splicing Events in BRCA

BRCA has four distinct molecular subtypes, known as Luminal-A, Luminal-B, Her-2, and Basal-like. We perform the principal component analysis (PCA) to characterize the expression pattern of SFs and ASs in the four molecular subtypes, as shown in Figures 1C,D. Notably, on the level of SFs, only Basal-like was clearly separated from the other three molecular subtypes. Besides, for AS-events, all four molecular-subtypes were clustered into more separated groups in the PCA plots, especially for the Basal-like subtype (Figures 1C,D). This finding indicated that the expression patterns of SFs and ASs were different across the four BRCA molecular subtypes. The type and the corresponding number of AS events in BRCA are shown in Figure 1E. Then, the survival-related SFs and ASs were determined by univariant Cox analysis in each molecular subtype, as shown in Figures 1F,H. However, the identified survival-related SFs and ASs showed little overlapping among the four subtypes (Figures 1G,I). This finding may indicate that the contribution of SFs and ASs in the progression of BRCA is distinct in the different molecular subtypes. The detailed results for the multivariable-Cox analysis of SFs and ASs were presented in Supplementary Tables S2, S3.

We perform KEGG pathway enrichment analysis to better describe the function of the identified survival-related ASs. We found that the identified ASs were closely related to several metabolic-related pathways, such as glycosaminoglycan biosynthesis and glycosphingolipid biosynthesis in Luminal A BRCA (Supplementary Figure S1A). In Luminal B BRCA, the survival-related ASs were enriched in DNA-repair related pathways, such as nonhomologous end-joining, and several metabolic-related pathways, such as thiamine metabolism and oxidative phosphorylation (Supplementary Figure S1B). Moreover, similar to the Luminal B BRCA, in Her-2 BRCA, survival-related ASs were enriched in DNA repair and metabolic-related pathways (Supplementary Figure S1C). In addition, survival-related ASs in Basal-like BRCA were enriched in metabolism- and autophagy-related pathways (Supplementary Figure S1D). Collectively, these results suggested that ASs may play important roles in BRCA, especially, each molecular subtype may be regulated by unique groups of SFs and ASs. Thus, the connections among SF, AS, and BRCA are subtypes specifically.

### Construction and Validation of Subtype-Specific Prognostic Risk Models Using Survival-Related SFs

**FIGURE 2:**
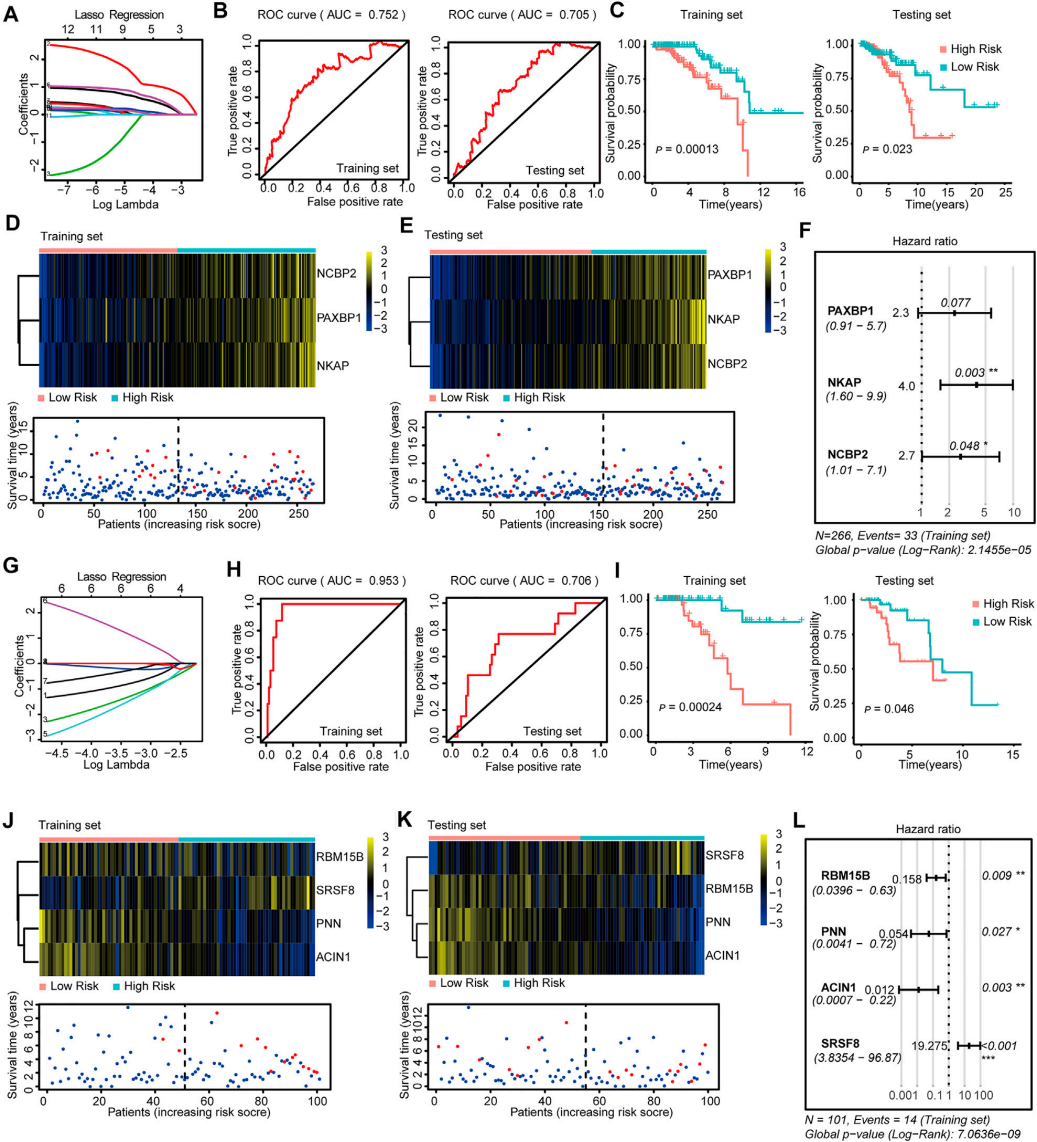
Construction and Validation of the SF-risk-models in Luminal-A and Luminal-B BRCA. **(A)** Lasso regression analysis of the top 20 survival-related SFs in Luminal-A BRCA. **(B)** Time-dependent ROC curve analyses showing AUC values (5 years) for the constructed SF-risk-models in Luminal-A BRCA. Left, training dataset right, testing dataset. **(C)** Kaplan-Meier plot showing the SF-risk-model of Luminal-A BRCA can accurately predict the patient’s prognosis (log-rank test). **(D)** Heatmap of the three key genes expression profiles in the training dataset of Luminal-A BRCA **(upper panel)**. Dot plots showing the survival time and RiskScore in the training set of Luminal-A BRCA (lower panel). **(E)** Heatmap of the three key genes expression profiles in the testing dataset of Luminal-A BRCA (upper panel). Dot plots showing the survival time and RiskScore in the testing dataset of Luminal-A BRCA **(lower panel)**. **(F)** Forest plot showing the multivariable Cox regression analysis of three key genes in SF-risk-model for Luminal-A BRCA. **(G)** Lasso regression analysis of the top 20 survival-related SFs in Luminal-B BRCA. **(H)** Time-dependent ROC curve analyses showing AUC values (5 years) for the constructed SF-risk-models in Luminal-B BRCA. Left, training dataset right, testing dataset. **(I)** Kaplan-Meier plot showing the SF-risk-model of Luminal-B BRCA can accurately predict the patient’s prognosis ((log-rank test)). **(J)** Heatmap of the four key genes expression profiles in the training dataset of Luminal-B BRCA **(upper panel)**. Dot plots showing the survival time and RiskScore in the training set of Luminal-A BRCA **(lower panel)**. **(K)** Heatmap of the four key genes expression profiles in the testing dataset of Luminal-B BRCA **(upper panel)**. Dot plots showing the survival time and RiskScore in the testing dataset of Luminal-A BRCA **(lower panel)**. **(L)** Forest plot showing the multivariable Cox regression analysis of four key genes in SF-risk-model in Luminal-B BRCA.

**FIGURE 3:**
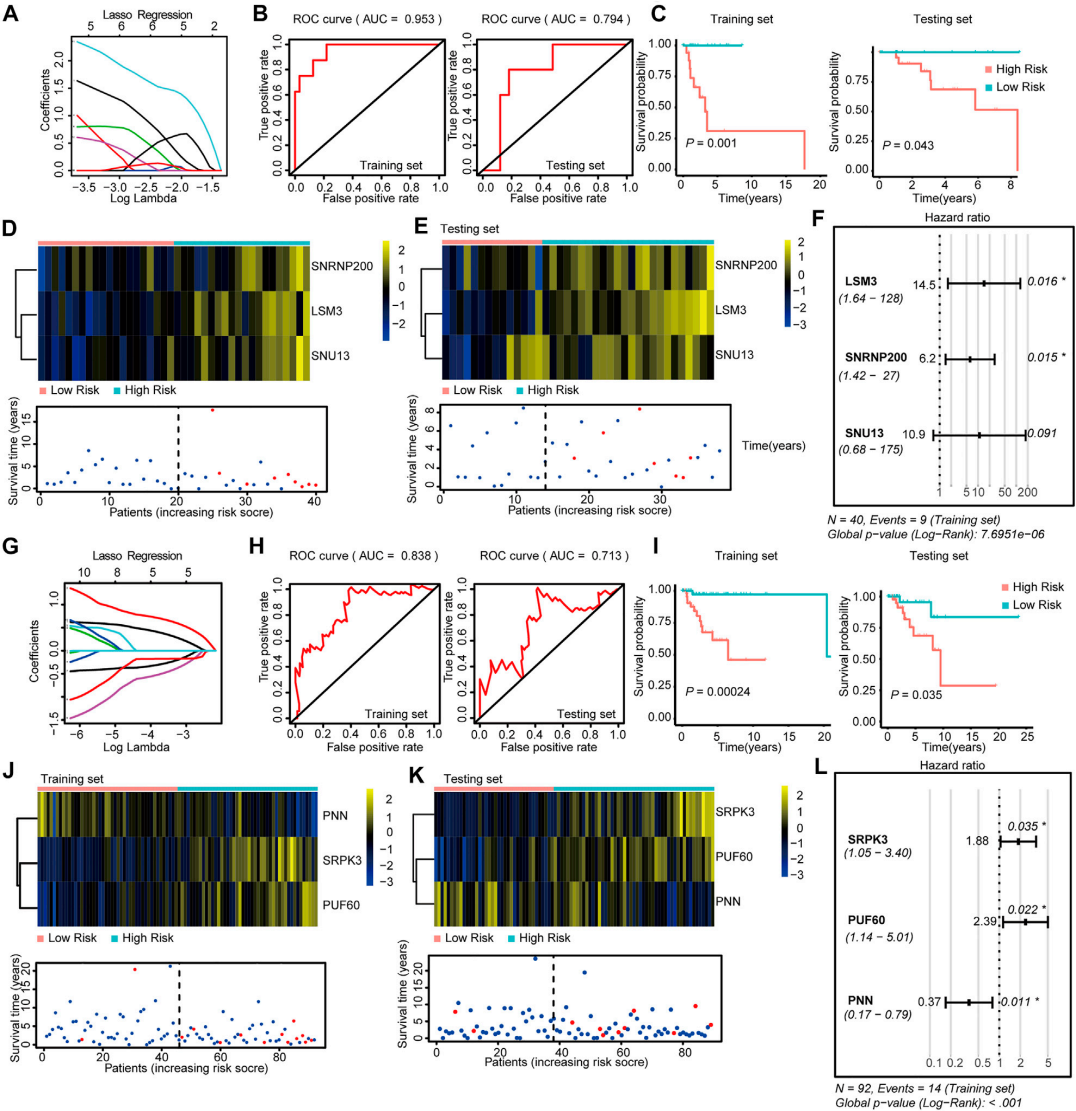
Construction and Validation of the SF-risk-models in Her-2 and Basal-like BRCA. **(A)** Lasso regression analysis of the top 20 survival-related SFs in Her-2 BRCA. **(B)** Time-dependent ROC curve analyses showing AUC values (5 years) for the constructed SF-risk-models in Her-2 BRCA. Left, training dataset right, testing dataset. **(C)** Kaplan-Meier plot showing the SF-risk-model of Her-2 BRCA can accurately predict the patient’s prognosis (log-rank test). **(D)** Heatmap of the three key genes expression profiles in the training dataset of Her-2 BRCA **(upper panel)**. Dot plots showing the survival time and RiskScore in the training set of Her-2 BRCA **(lower panel)**. **(E)** Heatmap of the three key genes expression profiles in the testing dataset of Her-2 BRCA **(upper panel)**. Dot plots showing the survival time and RiskScore in the testing dataset of Luminal-A BRCA **(lower panel)**. **(F)** Forest plot showing the multivariable Cox regression analysis of three key genes in SF-risk-model of Her-2 BRCA. **(G)** Lasso regression analysis of the top 20 survival-related SFs of Basal-like BRCA. **(H)** Time-dependent ROC curve analyses showing AUC values (5 years) for the constructed SF-risk-models in Basal-like BRCA. Left, training dataset right, testing dataset. **(I)** Kaplan-Meier plot showing the SF-risk-model of Basal-like BRCA can accurately predict the patient’s prognosis (log-rank test). **(J)** Heatmap of the four key genes expression profiles in the training dataset of Basal-like BRCA **(upper panel)**. Dot plots showing the survival time and riskscore in the training set of Basal-like BRCA **(lower panel)**. **(K)** Heatmap of the three key genes expression profiles in the testing dataset of Basal-like BRCA **(upper panel)**. Dot plots showing the survival time and RiskScore in the testing dataset of Basal-like BRCA **(lower panel)**. **(L)** Forest plot showing the multivariable Cox regression analysis of three key genes in SF-risk-model of Basal-like BRCA.

We constructed risk models in the four BRCA subtypes using Lasso-Cox regression to address the connection between survival-related SFs and prognosis. We initially generated training and testing datasets using TCGA-BRCA data and then subjected them to downstream prognosis model construction. We obtained four subtype-specific risk models for each molecular-subtype by using 12 SFs, and the RiskScores were calculated for each patient. The Lasso regressions for the risk model generation for Luminal-A, Luminal B, Her-2, and Basal-like subtype were shown in Figures 2A,G, 3A,G, respectively. The lambda selection for the lasso regression was shown in Supplementary Figure S1E. The results for univariable-Cox analysis of 12 SFs are shown in Table 1.

**TABLE 1:** Univariable Cox analysis of 12 selected SFs.

Gene symbol	HR	HR-95L	HR-95H	PAM50	*p* Value
*PAXBP1*	3.39	1.94	5.93	Luminal-A	<0.001
*NKAP*	3.30	1.87	5.85	Luminal-A	<0.001
*NCBP2*	2.43	1.41	4.21	Luminal-A	0.001
*RBM15B*	0.43	0.23	0.82	Luminal-B	0.010
*PNN*	0.53	0.31	0.88	Luminal-B	0.015
*ACIN1*	0.47	0.25	0.87	Luminal-B	0.016
*SRSF8*	1.82	1.12	2.98	Luminal-B	0.016
*LSM3*	11.30	2.94	43.40	Her-2	<0.001
*SNRNP200*	4.08	1.70	9.84	Her-2	0.002
*SNU13*	8.20	1.76	38.20	Her-2	0.007
*SRPK3*	1.78	1.21	2.63	Basal-like	0.003
*PUF60*	2.24	1.27	3.95	Basal-like	0.006
*PNN*	0.478	0.26	0.87	Basal-like	0.016

**Abbreviations:** HR, hazard ratio; HR-95 L/H, 95% confidence interval of the hazard ratio.

In Luminal A BRCA, a three-gene (*PAXBP1*, *NKAP*, and *NCBP2*) SF-risk-model was obtained with the area under curve (AUC) = 0.752 in the training set and AUC = 0.705 in the testing set (Figures 2A,B and Supplementary Figure S2A). The Kaplan–Meier survival analysis also showed good prognosis predicting power for this risk model in the training and testing datasets (Figure 2C). The three gene expression patterns and the RiskScore distribution of this risk model are shown in Figures 2D,E and Supplementary Figure S1E. In addition, the multivariable Cox analysis was performed, and the hazard ratio and the corresponding *p-*value are shown in Figure 2F. The Kaplan–Meier plots for individual genes involved in this risk model are shown in Supplementary Figure S2B.

The same data processing and analysis were also performed in other BRCA subtypes. We identified a four-gene SF-risk-model (*RBM15B*, *PNN*, *ACIN1*, and *SRSF8*) in Luminal-B BRCA with an AUC = 0.953 in the training set and AUC = 0.706 in the testing set (Figures 2G,H and Supplementary Figure S2A). The Kaplan–Meier plots also suggested good prognosis predicting power for this risk model in the training and testing datasets (Figure 2I). The expression pattern for the four-gene model and the RiskScore distribution are shown in Figures 2J,K and Supplementary Figure S1E. The hazard ratios and the corresponding *p* values for multivariable Cox analysis are shown in Figure 2L. The Kaplan–Meier plots for individual genes involved in this risk model are shown in Supplementary Figure S2C.

We also constructed a three-gene SF-risk-model (*LSM3*, *SNRNP200*, and *SNU13*) in patients with Her-2 BRCA with AUC = 0.953 in the training and AUC = 0.794 in the testing set (Figures 3A,B and Supplementary Figure S2A). A good prognosis predicting power was also suggested by the Kaplan-Meier analysis in the training and testing datasets (Figure 3C). The expression pattern for the three-gene model and the RiskScore distribution are shown in Figures 3D,E and Supplementary Figure S1E. The multivariable Cox analysis suggested that these genes were positive risk-related genes (Figure 3F). The Kaplan–Meier plots for individual genes involved in this SF risk model are shown in Supplementary Figure S2D.

Finally, we constructed a three-gene risk model (*SRPK3*, *PUF60*, and *PNN*) in Basal-like subtype with AUC = 0.838 in the training set and AUC = 0.713 in the testing dataset (Figures 3G,H and Supplementary Figure S2A). The Kaplan-Meier analysis also suggested that this risk model can accurately predict the prognosis of patients with Basal-like BRCA (Figure 3I). The expression pattern and the RiskScore distribution of this three-gene risk model are shown in Figures 3J,K and Supplementary Figure S1E. Among these genes, SRPK3 and PUF60 were identified as positive risk-related genes, whereas PNN was recognized as a negative risk-related gene (Figure 3L). The Kaplan–Meier plots for individual genes involved in this SF-risk-model are shown in Supplementary Figure S2D. The figure shows that most of the selected SFs are correlated with the overall survival of BRCA patients. In addition, the clinical relevance of SFs in Luminal-A and Luminal-B BRCA was validated using a combination of GEO datasets by KM-plotter database (http://kmplot.com/). Consistently, all six SFs showed high correlations between mRNA expression and PFS (Supplementary Figures S3A,B).

**FIGURE 4:**
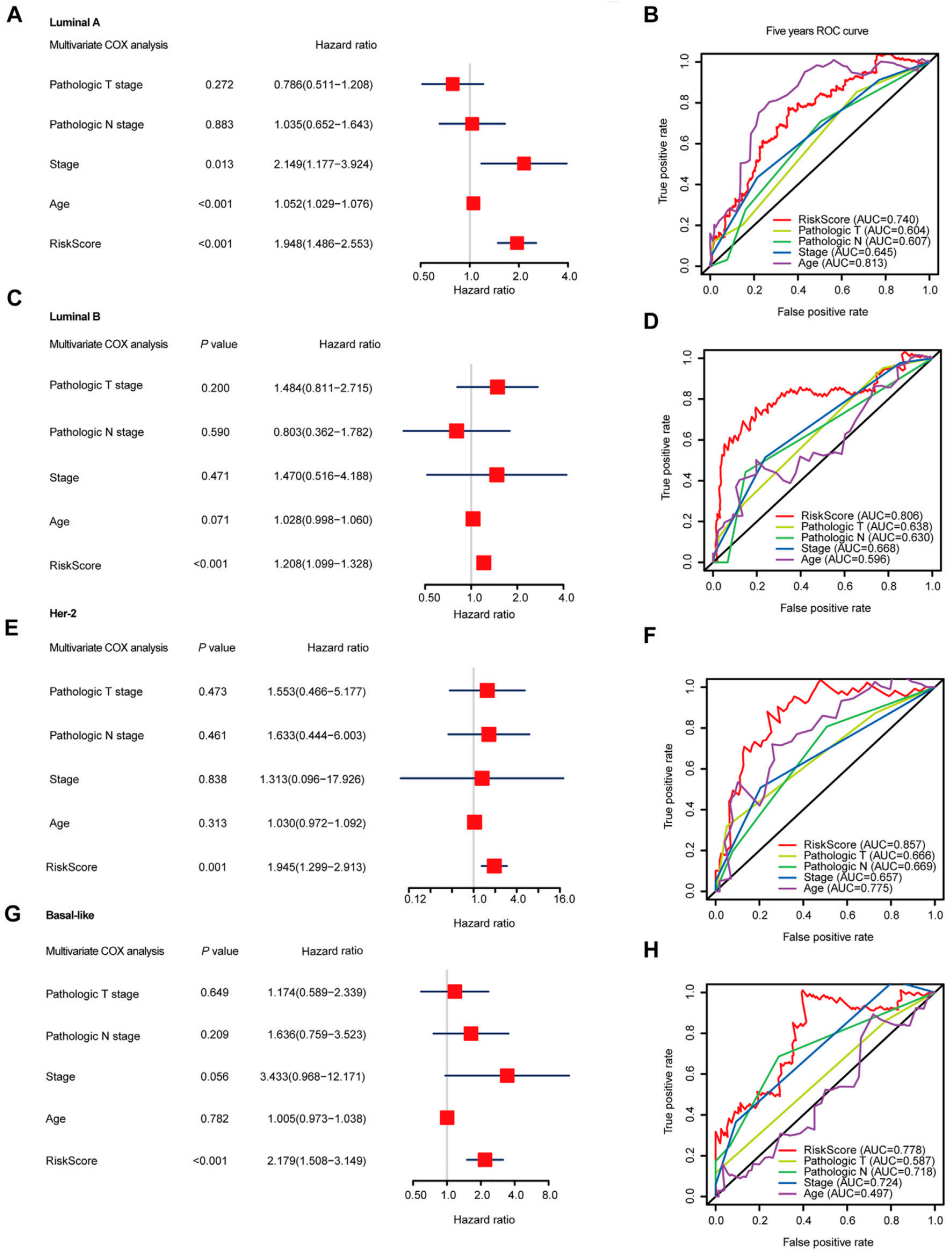
Multivariable Cox regression analyses and ROC analyses of SF-risk-models in Luminal, Her-2, and Basal-like BRCA. **(A)** Forest plots showing the multivariable Cox regression analyses of the SF-risk-models of Luminal-A BRCA. **(B)** Time-dependent ROC analysis (5 years AUC was shown) of SF-risk-models and other clinical-parameters in Luminal-A BRCA. **(C)** Forest plots showing the multivariable Cox regression analyses of the SF-risk-models of Luminal-B BRCA. **(D)** Time-dependent ROC analysis (5 years AUC was shown) of SF-risk-models and other clinical-parameters in Luminal-B BRCA. **(E)** Forest plots showing the multivariable Cox regression analyses of the SF-risk-models of Her-2 BRCA. **(F)** Time-dependent ROC analysis (5 years AUC was shown) of SF-risk-models and other clinical-parameters in Her-2 BRCA. **(G)** Forest plots showing the multivariable Cox regression analyses of the SF-risk-models of Basal-like BRCA. **(H)** Time-dependent ROC analysis (5 years AUC was shown) of SF-risk-models and other clinical-parameters in Basal-like BRCA.

To further evaluate the prognosis-predicting performance of the SF-riskmodels for each BRCA subtype, we perform multivariable-Cox analysis and time-dependent ROC analysis by using the model-predicted RiskScore and other clinical parameters. The multivariate Cox analysis indicated that the predicted RiskScore could independently predict the OS for the four types of BRCA (Figures 4A,C,E,G). In Luminal-A BRCA, the predicted RiskScore has an AUC = 0.740, which is lower than the patient’s age (AUC = 0.813), as shown in Figure 4B. Particularly, the RiskScore showed better predicting performance in Luminal-B, Her-2, and Basal-like BRCA than in other well-established clinical parameters (Figures 4D,F,H). These results suggest that the four subtype-specific risk models can be used independently to predict the OS in BRCA patients with different molecular subtypes.

### Mechanistic Exploration of Model-Predicted High-Risk Patients by Gene Set Enrichment Analysis

The results indicated that the SF-RiskScore could independently and accurately predict the prognosis of BRCA patients. We subsequently used gene set enrichment analysis (GSEA) to explore the possible mechanisms that linked the RiskScore and prognosis. The GSEAs were performed using model predicted low- and high-risk samples to calculate the fold change of gene expression, and the analysis was conducted using Hallmark 50 and KEGG pathway datasets. The detailed analysis results for GSEA analysis are shown in Supplementary Tables S4, S5.

**FIGURE 5:**
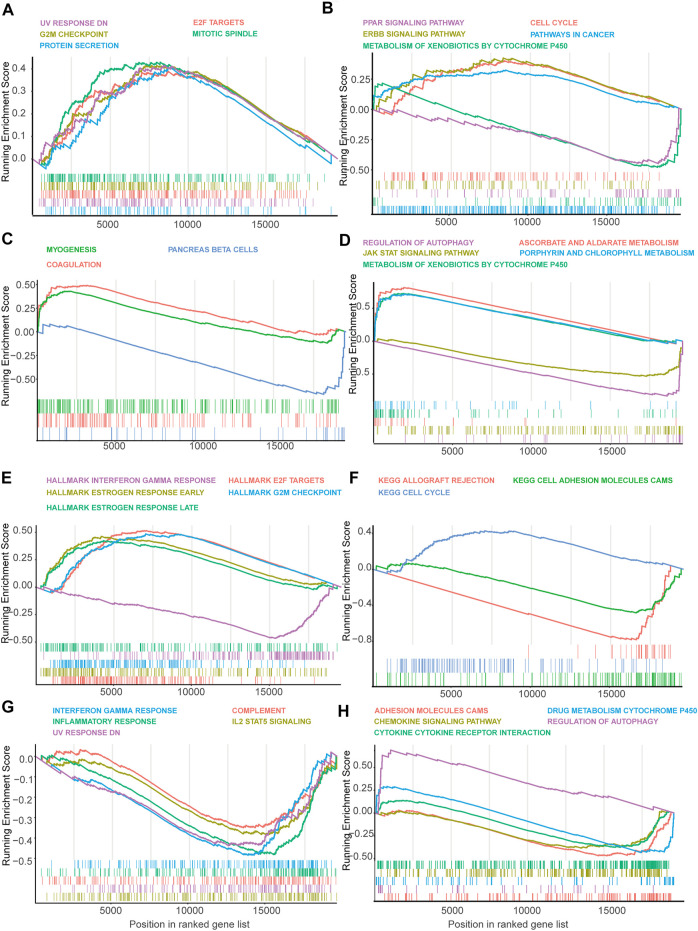
Gene set enrichment analysis (GSEA) of genes in high-risk and low-risk patients in Luminal, Her-2 and Basal-like BRCA predicted by SF-risk-model. **(A,B)** Gene set enrichment analysis (GSEA) showing the enrichment of Hallmarks **(A)** and KEGG pathways **(B)** in high-risk and low-risk patients with Luminal-A BRCA. **(C,D)** GSEA showing the enrichment of Hallmarks **(C)** and KEGG pathways **(D)** in high-risk and low-risk patients with Luminal-B BRCA. **(E,F)** GSEA showing the enrichment of Hallmarks **(E)** and KEGG pathways **(F)** in high-risk and low-risk patients with Her-2 BRCA. **(G,H)** GSEA showing the enrichment of Hallmarks **(G)** and KEGG pathways **(H)** in high-risk and low-risk patients with Basal-like BRCA.

In the Luminal A subtype, the GSEA revealed that high-risk samples were enriched in genes related to cell cycle progression, such as G2M checkpoint and mitotic spindle (Figure 5A). Besides, GSEA using KEGG gene set suggested that high-risk patients were upregulated with cell cycle and ERBB signaling-related genes (Figure 5B). However, PAPP signaling-related genes and cytochrome P450 pathway-related genes were enriched in low-risk patients, indicating that the signaling may have a protective function in Luminal A BRCA (Figure 5B).

In Luminal B BRCA, we found that the top enriched Hallmark gene sets were not closely related to cancer progression (Figure 5C). However, GESA suggested that Aldarate metabolism- and chlorophyll metabolism-related genes were enriched in high-risk patients, indicating that these genes were negatively correlated with the prognosis (Figure 5D). Interestingly, cytochrome p450 related genes, which were enriched in the low-risk Luminal A BRCA, were enriched in the high-risk Luminal B BRCA, suggesting that this set of genes may play a different role in the two Luminal BRCA subtypes (Figure 5D).

In Her-2 BRCA, as expected, estrogen response-related genes were enriched in high-risk patients (Figure 5E). Similar to the Luminal A BRCA (Figure 5A), the E2F target genes and G2M checkpoint-related genes were also enriched in the high-risk patients (Figure 5E). Notably, interferon-gamma response-related genes were enriched in low-risk patients, suggesting that this signaling may have a protective effect in Her-2 BRCA (Figure 5E). In addition, GSEA using KEGG gene set showed that cell cycle-related genes were enriched in high-risk patients, and adhesion molecule CAM-related genes seem to have a protective function in Her-2 BRCA (Figure 5F).

In Basal-like BRCA, we found that interferon-gamma-related genes, inflammatory response-related genes, and IL-2 STAT5 signaling related genes were enriched in low-risk patients (Figure 5G); while, autophagy-related genes, were enriched in high-risk patients (Figure 5H). Collectively, GSEA suggested that many cancer-related signaling may be linked with SFs to regulate the progression of BRCA.

### Comprehensive Analysis of Genes in Subtype-Specific SF-Risk-Models

**FIGURE 6:**
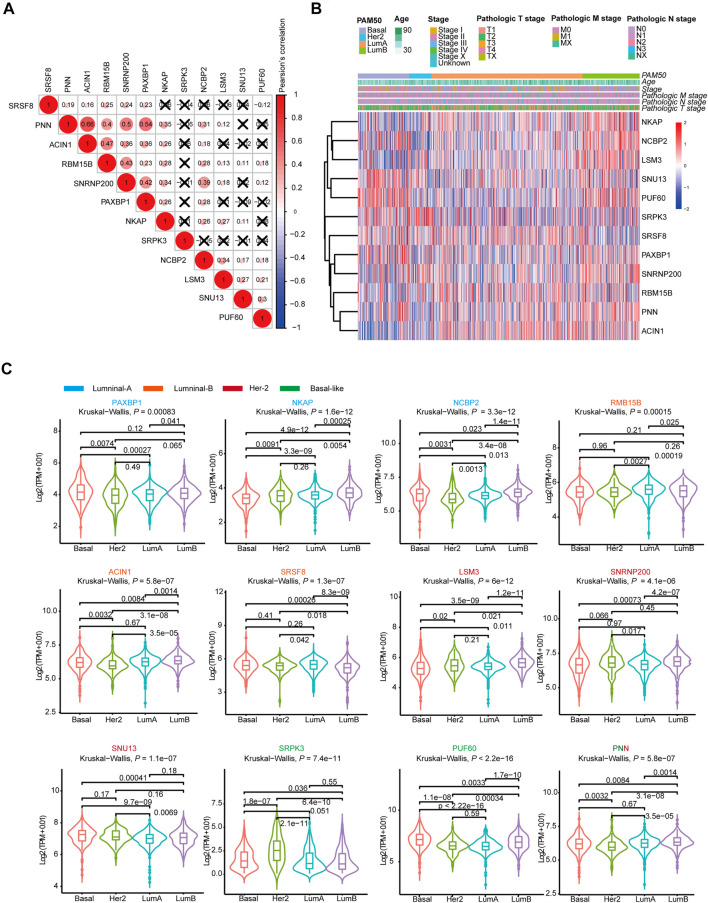
The mRNA expression landscape of 12 selected SFs in SF-risk-models. **(A)** Clustered heatmap showing the correlation of genes expression in four SF-risk-models. The correlation was calculated by Pearson’s correlation using log2 (TPM+0.01). Not statistically significant correlations were defined as *p* > 0.05 and marked by a black cross. **(B)** Clustered heatmap showing the genes expression and clinical information in BRCA patients. **(C)** Violin plots showing identified gene expression in Luminal-A, Luminal-B, Her-2 and Basal-like BRCA. The *p* values were calculated by Wilcox-test (two groups comparison) and Kruskal- Wallis test (four groups comparison).

**FIGURE 7:**
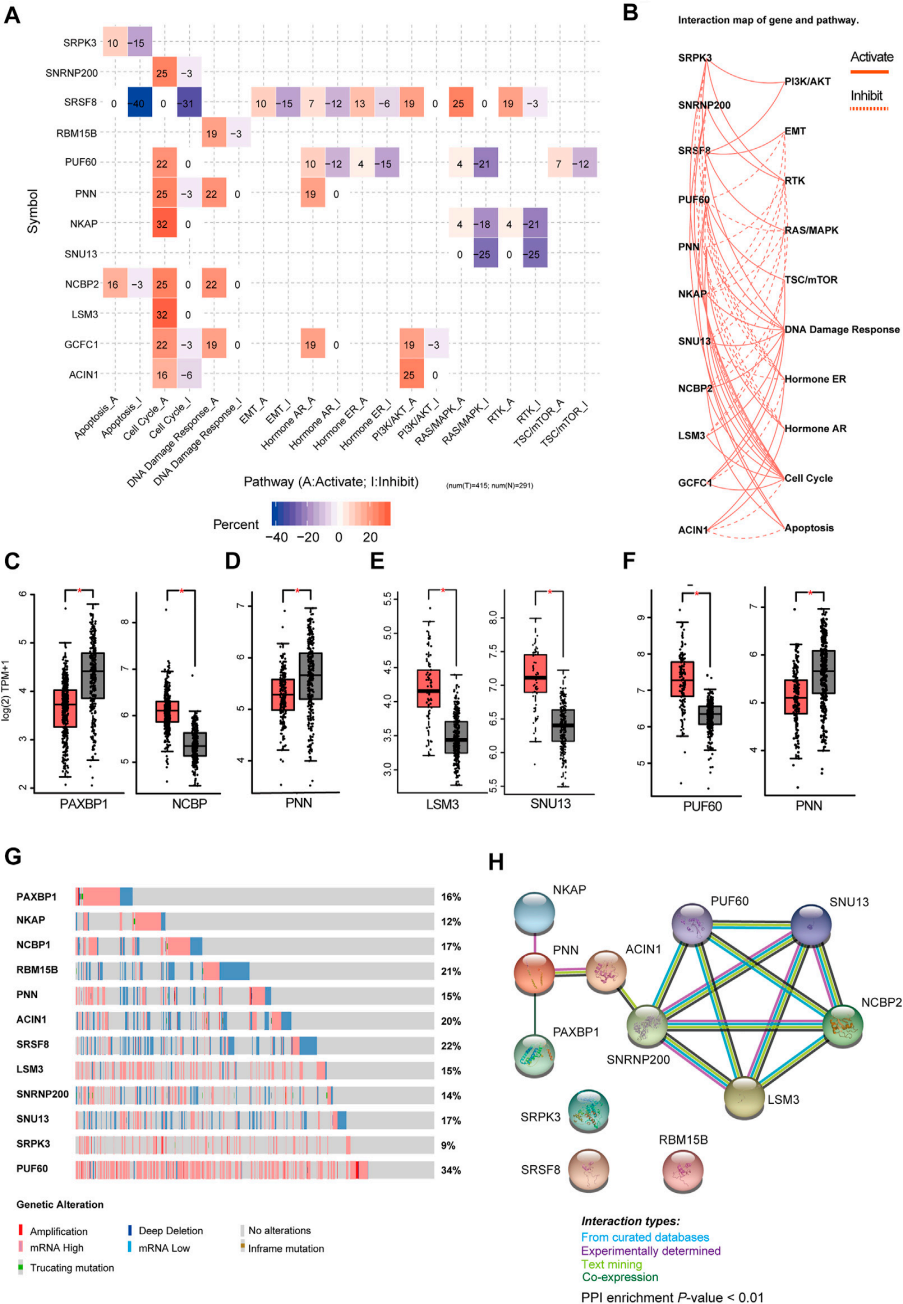
Comprehensive analysis of genes in SF-risk-models. **(A)** Heatmap showing the correlation between the 12 selected genes and the tumor-essential pathways. **(B)** Connection network showing the relationship between 12 selected genes and the tumor-essential pathways. **(C–F)** Boxplots showing the significant differentially expressed SFs in Luminal-A **(C)**, Luminal-B **(D)**, Her-2 **(E)** and Basal-like **(F)** BRCA. **(G)** OncoPrint showing the copy number alterations and mRNA expression alterations of 12 SFs in SF-risk-models. The analysis was performed by cBioProtal database. **(H)** Protein-protein interaction (PPI) analysis of genes in SF-risk-models by STRING database.

**FIGURE 8:**
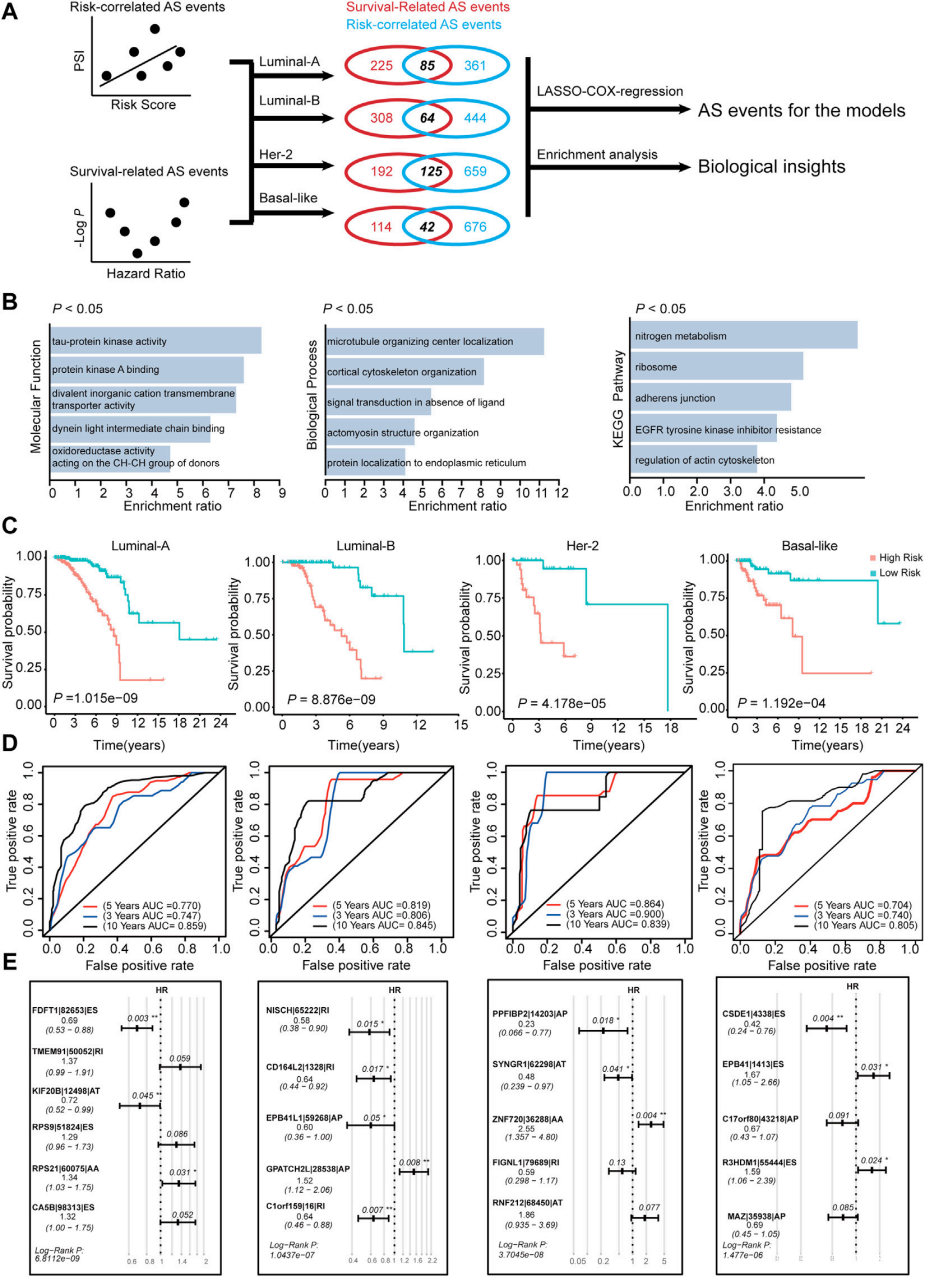
Construction of AS-risk-models in four subtypes of BRCA using AS-events. **(A)** The workflow of construction the AS-risk-models. **(B)** The gene ontology analysis of selected AS-events which are shared by correlation-significant ASs and survival-significant-ASs. **(C)** Kaplan-Meier plot showing the AS-risk-models can accurately predict the patient’s prognosis (log-rank test). **(D)** Time-dependent ROC curve analyses showing AUC values for the constructed AS-risk-models in four types of BRCA. **(E)** Forest plots showing the multivariable Cox regression analysis of key genes in SF-risk-models of four types of BRCA.

Then, we analyzed the genes expressed in the four subtype-specific risk-models using different approaches. The correlation analysis showed that some genes in the risk-models were positively correlated at the mRNA level, indicating intricate regulatory cascade may exist between these SFs (Figure 6A). The *p*-values for the correlation analysis were shown in Supplementary Table S6. Furthermore, the links among molecular subtypes, clinical parameters, and the mRNA expression of the 12 risk-model-related genes were shown by clustered heatmap (Figure 6B). In addition, violin plots showed that most 12 risk model-related genes were differentially expressed among four molecular subtypes of BRCA (Figure 6C). To further describe the line between the 12 selected genes and cancer-essential pathways, we analyze these genes by using the GSCALite database (http://bioinfo.life.hust.edu.cn/web/GSCALite/). We found that most genes were positively correlated to the cell cycle (Figure 7A). *SRSF8*, which is negatively correlated with the prognosis of Luminal B BRCA, was strongly negatively correlated with apoptosis and cell cycle (Figure 7A). The interaction network of the analyzed genes and pathways is shown in Figure 8B. Interestingly, pathway analysis showed that *SRSF8* was involved in 7 out of 10 analyzed pathways, thereby suggesting that it has intrinsic biological functions in BRCA (Figures 7A,B).

Furthermore, we analyzed the mRNA expression of the 12 risk-related genes using GEPIA database (http://gepia2.cancer-pku.cn/) in four molecular subtypes of BRCA. We found that *PAXBP1* and *NCBP* were differentially expressed in Luminal A BRCA compared with normal samples (Figure 7C). However, although *PAXBP1* is downregulated in Luminal A BRCA, it has been shown as a good prognosis gene (Supplementary Figure S2A). In Luminal B BRCA, *PNN* was the only gene that passed the significant threshold (log FC > 0.5, *p* < 0.01) and was downregulated in tumor samples (Figure 7D). In Her-2 BRCA, *LSM3* and *SNU13* were significantly upregulated in tumor samples and both genes were negatively correlated with prognosis (Figure 7E, Supplementary Figure S2C). In Basal-like BRCA, *PUF60* was upregulated and *PNN* was downregulated in tumor samples. This finding was consistent with the survival analysis that *PUF60* was a poor prognosis gene, and *PNN* was positively correlated with the prognosis (Figure 7F, Supplementary Figure S2D). However, although some SFs in the SF risk models showed a significant correlation with clinical outcome, the mRNA expression was comparable between tumor and normal tissue (Supplementary Figures S4A–D).

The mRNA expression alternation and gene mutation pattern for the 12 SFs were also analyzed by Oncoprint using cBioPotal database. The results showed that high mRNA expression alteration existed in all 12 analyzed SFs (Figure 7G). Notably, *PUF60* showed a high frequency of mRNA hyperexpression among BRCA samples. However, only a small fraction of samples were involved in gene amplification, deletion, and mutation. This finding indicated that genetic level alterations were not the primary driving force for the dysregulation of these SFs (Figure 7G). To further address the relationship between the identified 12 SFs, the protein-protein interaction (PPI) network was visualized using the STRING database. The PPI network indicated that *LSM3*, *SNRNP200*, *PUF60*, *NHP2L1*, and *NCBP2* were closely interconnected, suggesting a potential regulatory cascade among these proteins (Figure 7H). The analysis showed that *SRPK3*, *SRSF8*, and *RBM15B* have no connection with other genes, indicating that these proteins may function independently in tumors (Figure 7G). In summary, these results suggested that the 12 genes selected for risk prediction have strong connections to the tumor-related pathways. In addition, genes that are differentially expressed between the tumor and normal tissue indicated that they may serve as reliable markers to predict a patient’s disease outcome.

### Identification of Prognosis-Related AS-Events Using SF-Risk-Models

We have constructed risk models for different molecular subtypes of BRCA by using SFs. Considering that AS events are regulated mostly by SFs, we identified prognosis-related AS events using the constructed SF models. The workflow for identification of the prognosis-related AS events is shown in Figure 8A. We initially calculated the correlation between RiskScore (calculated by SF-risk-models) and all PSI values of AS events. In addition, the survival-related AS events were screened using univariable Cox analysis. Then, the survival-related AS events and the risk-correlated AS events were combined, and the intersected AS events (85 in Luminal A, 64 in Luminal B, 125 in Her-2, and 42 in basal-like) were screened out and subjected to downstream LASSO-Cox regression analysis and risk model construction (Figure 8A). In addition, the intersected AS events were subjected to enrichment analysis to explore their biological functions. Enrichment analysis suggested that these AS events were related to microtubule organizing center localization, signal transduction in the absence of ligand, adherence junction, EGFR tyrosine kinase inhibitor resistance, and regulation of actin cytoskeleton. Thus, these identified AS events may play important roles in tumor progression (Figure 8B). Interestingly, AT was enriched in Luminal A BRCA, whereas ES was enriched in Her-2 and Basal-like BRCA, besides, RI was enriched in Luminal B BRCA (Supplementary Figures S6A,B and Supplementary Table S7). This result indicated different molecular-subtype of BRCA may have their unique alternative-splicing preference to regulating its progression.

Subsequently, we constructed risk models using the intersected AS event identified in Figure 8A. In Luminal A, Luminal B, Her-2, and Basal-like BRCA, four risk models were identified using AS events via LASSO regression and multivariable Cox analysis (Supplementary Figure S5A). We identified 6, 5, 5, and 5 key AS events in Luminal-A, Luminal-B, Her-2, and Basal-like AS-risk-model, respectively (Figure 8E). The Oncoprint analysis of the genes involved in the final AS models suggested that they may manifest moderate levels of genetic alterations, such as amplification, deep deletion and mutations (Supplementary Figure S5C). We also analyzed the mRNA expression levels of these genes between tumor and normal tissue in the matched BRCA subtypes; however, only a few genes were dysregulated (Supplementary Figure S5D), we think that these genes may contribute to cancer progression, not at the absolute-expression level. The detailed information and splicing patterns for identified AS events in the AS risk models are shown in Supplementary Figure S7.

Subsequently, the patients were grouped into high- and low-risk groups by using the calculated AS-RiskScore, and the Kaplan-Meier analysis showed that the AS-risk-models can accurately predict the patient’s prognosis (Figure 8C). Furthermore, ROC analysis showed that the constructed models have good performance in the prediction of three-, five-, and 10 years OS of BRCA patients (Figure 8D). In addition, the RiskScores (SF-RiskScore and AS-RiskScore) predicted by the SF-risk-models and AS-risk-models are significantly correlated. This finding indicated that the prediction power of the two types of models was highly consistent (Supplementary Figure S5B). Importantly, multivariable Cox analysis indicated that the AS events involved in the AS risk models were independently correlated with the OS of BRCA patients (Figure 8E). In summary, we combined the SF risk models, survival-related AS events, and LASSO-Cox analysis to construct four subtype-specific AS models. The ROC and Kaplan-Meier analysis suggested that both models have good robustness and accuracy to predict the prognosis of BRCA patients with different molecular subtypes.

## Discussion

Dysregulation of gene expression has been associated with tumor initialization and progression. However, due to the complexity of mRNA maturation and processing and the existence of alternative splicing, a new layer of regulation is added; thus, tumor biology is beyond the well-known “central dogma” (Lee and Abdel-Wahab, 2016). Emerging evidence suggests that SFs play important roles in tumors. SFs are mainly RNA binding proteins, which are involved in the mRNA transcription and processing and ultimately regulates the isoform-specific protein synthesis (Naro et al., 2015). SF controlled AS events implicated in various tumor-related biological processes, such as cell cycle progression, sustaining activation of growth/survival signaling, reprogramming of tumor-specific metabolic processes, as well as evading the immune surveillance (Ding et al., 2020). Thus, the abnormal alternative splicing has been recognized as new cancer “hallmark.”

Alternative splicing events are mainly controlled and processed by SFs, and alteration of SF expression has been observed in many types of cancer, including BRCA (Sveen et al., 2016). The function of SFs has been recognized as highly context-dependent, and evidence suggests that some SFs switch between oncogenes and tumor suppressors in different malignancies (Shchelkunova et al., 2013). SFs have been found generally dysregulated in cancer, and the number of differentially regulated SFs (tumor versus normal) is substantially different among different malignancies (Sveen et al., 2016). Evidence suggests that BRCA is more associated with cancer-specific AS-events than with other cancer types (Dvinge and Bradley, 2015). Aberration of AS pattern has been frequently detected in BRCA. However, the upstream regulators that control the tumor-specific AS event lack systemic characterization. We found that the expression patterns of SFs and ASs are distinct in different molecular subtypes of BRCA, suggesting that subtype-specific SF–AS regulatory networks may exist. This hypothesis is further supported by the Cox analysis of SFs and ASs. It showed that very few survival-related SFs and ASs are shared by multiple BRCA subtypes. Interestingly, PCA showed that the Basal-like BRCA has a different expression pattern in SFs and ASs. This finding suggests that SFs and ASs may play different functional roles in Basal-like BRCA compared with others.

We constructed four subtype-specific SF risk models for each BRCA subtype, and 12 SFs were involved in the model construction. Interestingly, correlation analysis showed that most SFs were positively correlated at the mRNA level. The PPI analysis also suggested that the identified SFs were highly interconnected. Interestingly, we also found that all the 12 SFs were differentially expressed across at least two different PAM50 subtypes (Figure 6C), indicating some SFs may regulate the progression of BRCA in a subtype-specific manner. *PUF60* has been identified that involved the progression of Basal-like MDA-MB-231 cells by regulating PTEN signaling (Sun et al., 2019b). We also speculated that these SFs may act synergistically to regulate the downstream AS events and finally modulate the behavior of tumor cells. *SRSF8*, which is a member of serine- and arginine-rich SF, was related to multiple pathways in the pathway analysis result. Although little direct evidence shows connections between *SRSF8* and cancer, our *in silico* analysis suggests that it may be valuable for deep research. Besides, GSEA indicates that interferon-gamma pathway plays a protective role in Her-2 and Basal-like BRCA. The cytochrome P450 pathway seems to be involved in Luminal A/B BRCA, but its functional role may be highly context-dependent. We speculate that SFs control several tumor-essential signaling. However, this regulatory role may be more dependent on systemic effects caused by the dysregulation of SFs but not the change of some key AS events.

Some studies developed risk-predicting models using AS events and showed good prognosis predicting potential (Zhang et al., 2019b; Liu et al., 2020; Yu et al., 2020). However, considering that BRCA is a highly heterogeneous malignancy with different molecular subtypes that display distinct biological properties, and thus identify that biomarkers specific to each subtype are more applicable for clinical usage. In addition, although deep RNA-sequencing provides valuable insight into the AS process, the precise quantification of AS events are challenging, thereby influencing the robustness of the downstream analysis (Sveen et al., 2016). To address this challenge, we constructed four risk prediction models using the survival-related SFs in each BRCA subtype, and the downstream analysis suggested that the models can accurately predicate the prognosis of BRCA patients. Among these models, 12 risk-related SFs were identified, and the following comprehensive analysis indicates that these genes may play essential roles in BRCA progression and diagnosis. Then, the AS events that are significantly correlated with the RiskScore predicted by the SF-risk-models were screened out and combined with univariable Cox analysis to pinpoint the survival significant AS events. Finally, we constructed four subtype-specific risk models using the selected AS events and the following ROC analysis showed optimistic risk-predicting power. The combination of SF-risk-models and survival-related AS events may improve the overall robustness of the constructed AS risk models. However, due to the limitation of available data in the TCGA database, the SF-risk-models and AS-risk-models need to be optimized and validated using more datasets, and the predicting power should be tested in local BRCA cohorts.

In summary, we systemically analyze the expression landscape and clinical relevance of SFs and ASs by interpreting the mRNA expression and AS event data. Four SF-risk-models and four AS-risk-models were constructed for each molecular subtype of BRCA. The identified SFs and ASs may serve as targets for the treatment and intervention of BRCA. However, the main limitation of this study is that the data used were obtained from several public databases. Therefore, the findings need to be validated in future clinical trials. In addition, although we described the potential mechanistic links between the SF risk models and BRCA, the actual connections still need to be verified by experimental approaches.

## Conclusion

To sum up, our study shows that the SFs and ASs have promising potential as biomarkers and therapeutic targets for diagnosis and prognosis in BRCA. The constructed risk-predicting models have good performance in predicting the prognosis of BRCA patients.

## Data Availability

The datasets presented in this study can be found in online repositories. The names of the repository/repositories and accession number(s) can be found in the article/Supplementary Material.

## Abbreviations

SF, splicing factor; AS, alternative splicing; BRCA, breast cancer; TCGA, the cancer genome atlas; ROC, receiver operating characteristic; AUC, area under curve; AA, alternate acceptor site; AD, alternate donor site; AP, alternate promoter; AT, alternate terminator; ES, exon skip; ME, mutually exclusive exons; RI, retention intron; TPM, transcripts per million; PSI, percent spliced index; PCA, principal component analysis; PPI, protein-protein interaction; GSEA, gene set enrichment analysis; NES, normalized enrichment score.
