# Supplementary figures and images for: Comprehensive Analysis of Splicing Factor and Alternative Splicing Event to Construct Subtype-Specific Prognosis-Predicting Models for Breast Cancer
Source: Front Genet. 2021 Sep 24;12:736423. doi: 10.3389/fgene.2021.736423 (PMC8497829; doi:10.3389/fgene.2021.736423)

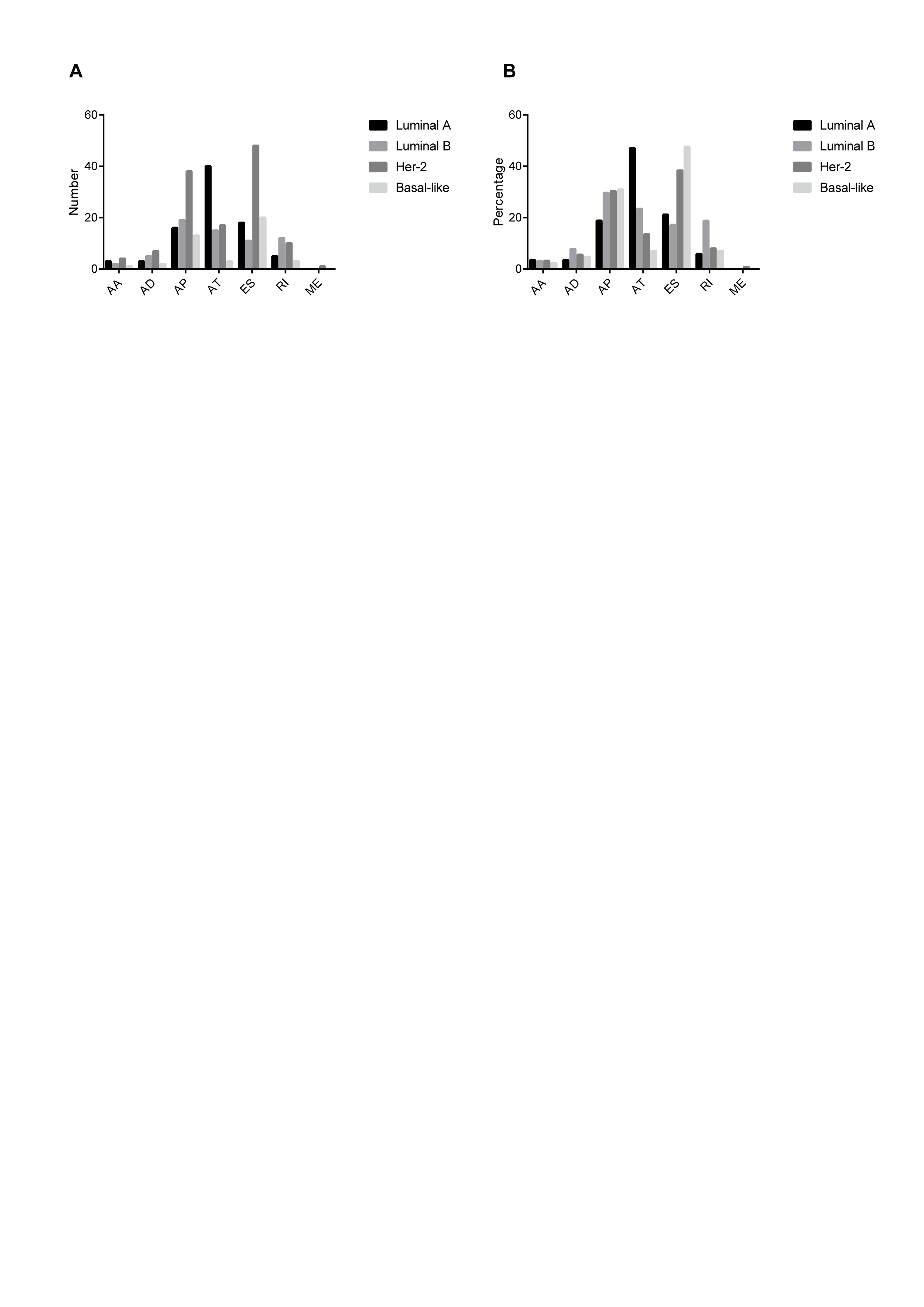

Supplement: Supplementary file 3 [file Image6.TIF]

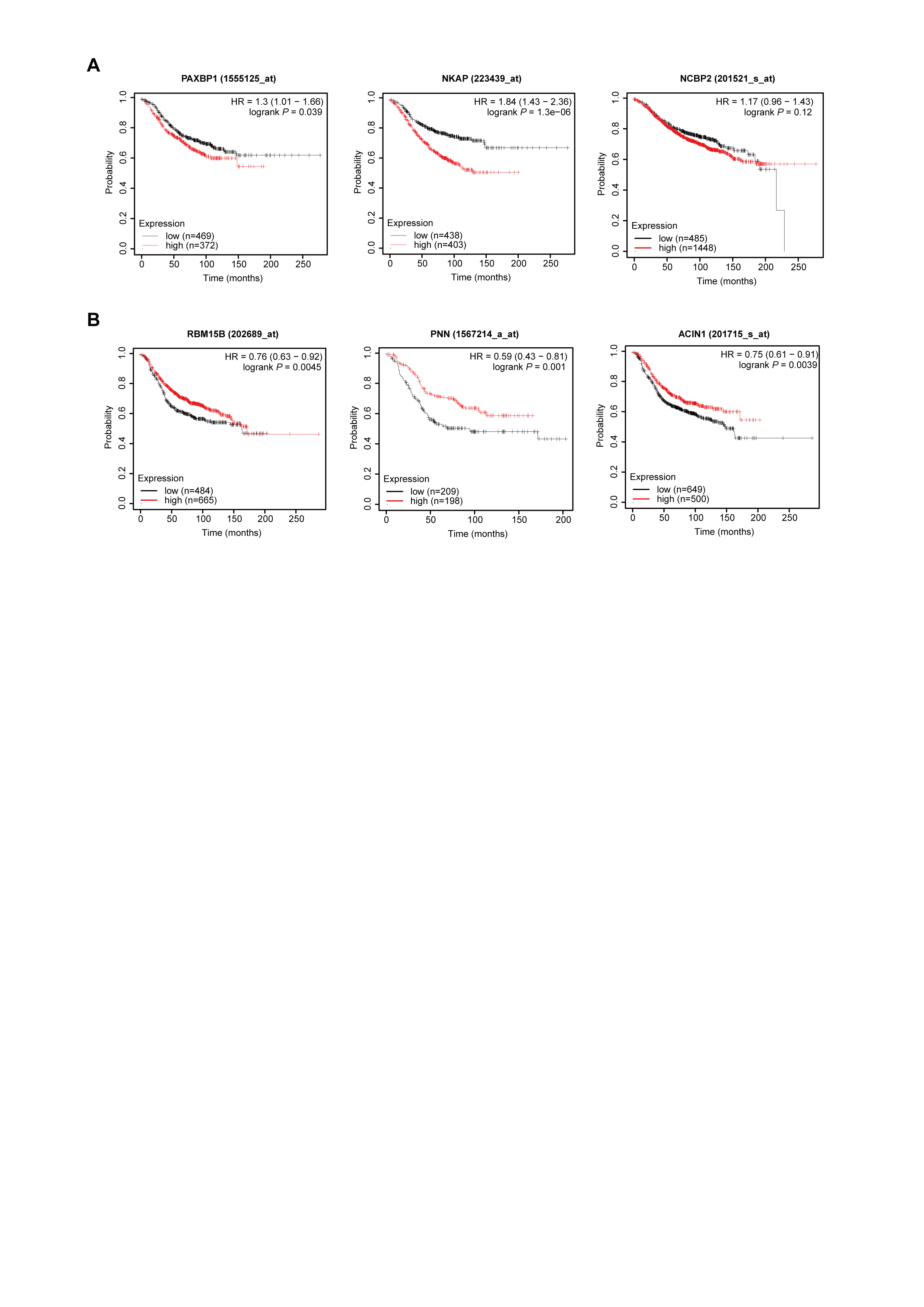

Supplement: Supplementary file 4 [file Image3.TIF]

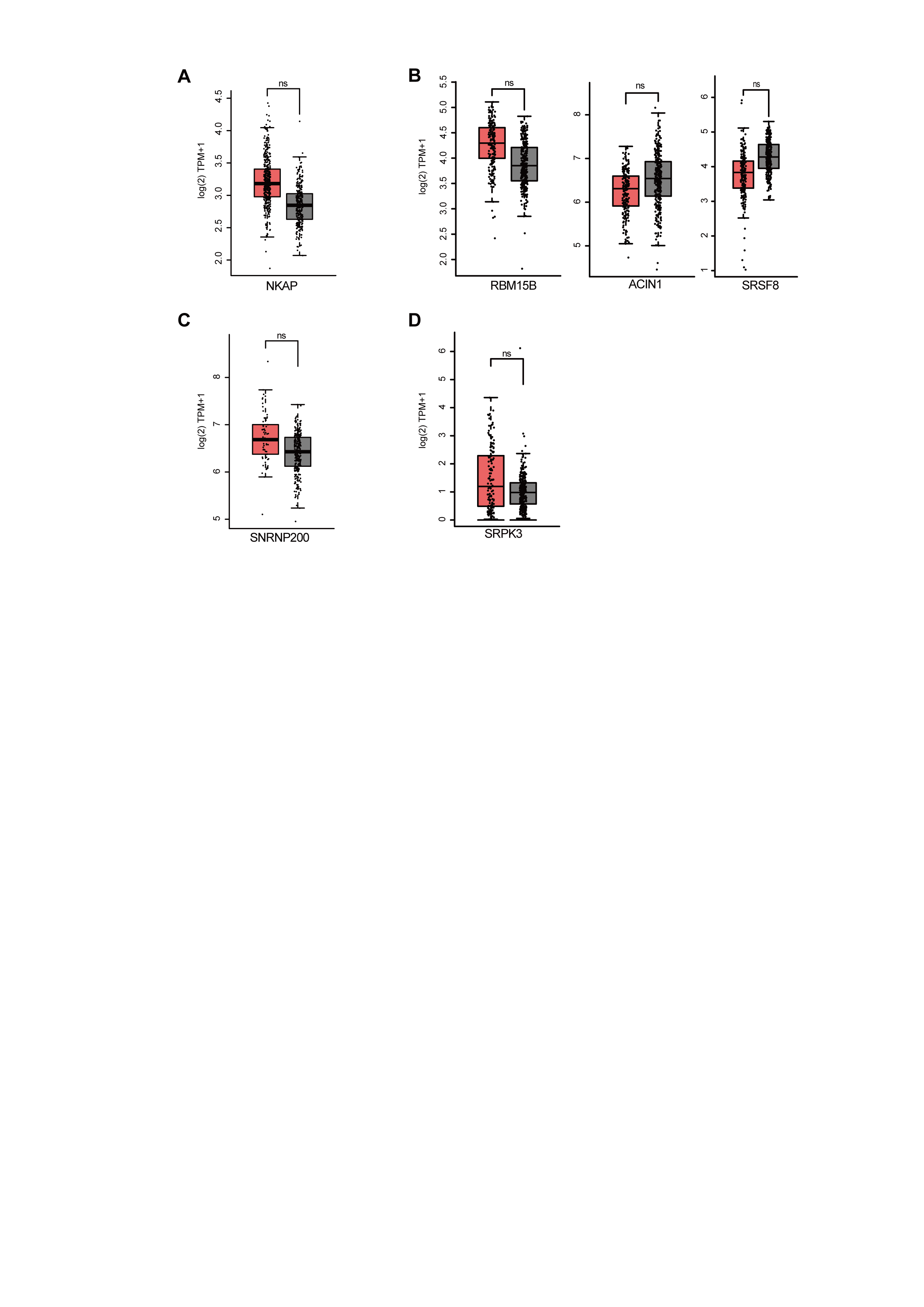

Supplement: Supplementary file 5 [file Image4.TIF]

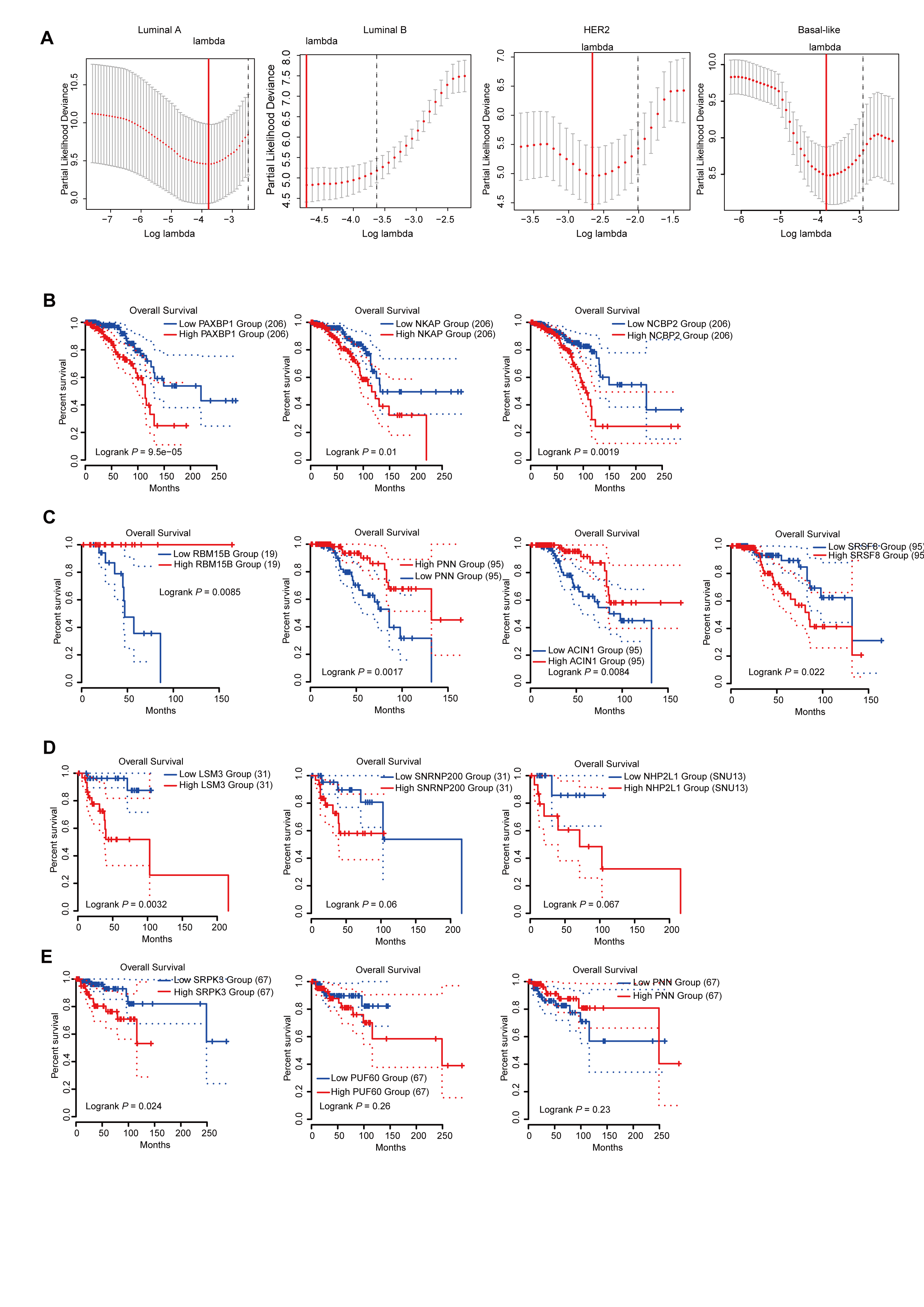

Supplement: Supplementary file 6 [file Image2.TIF]

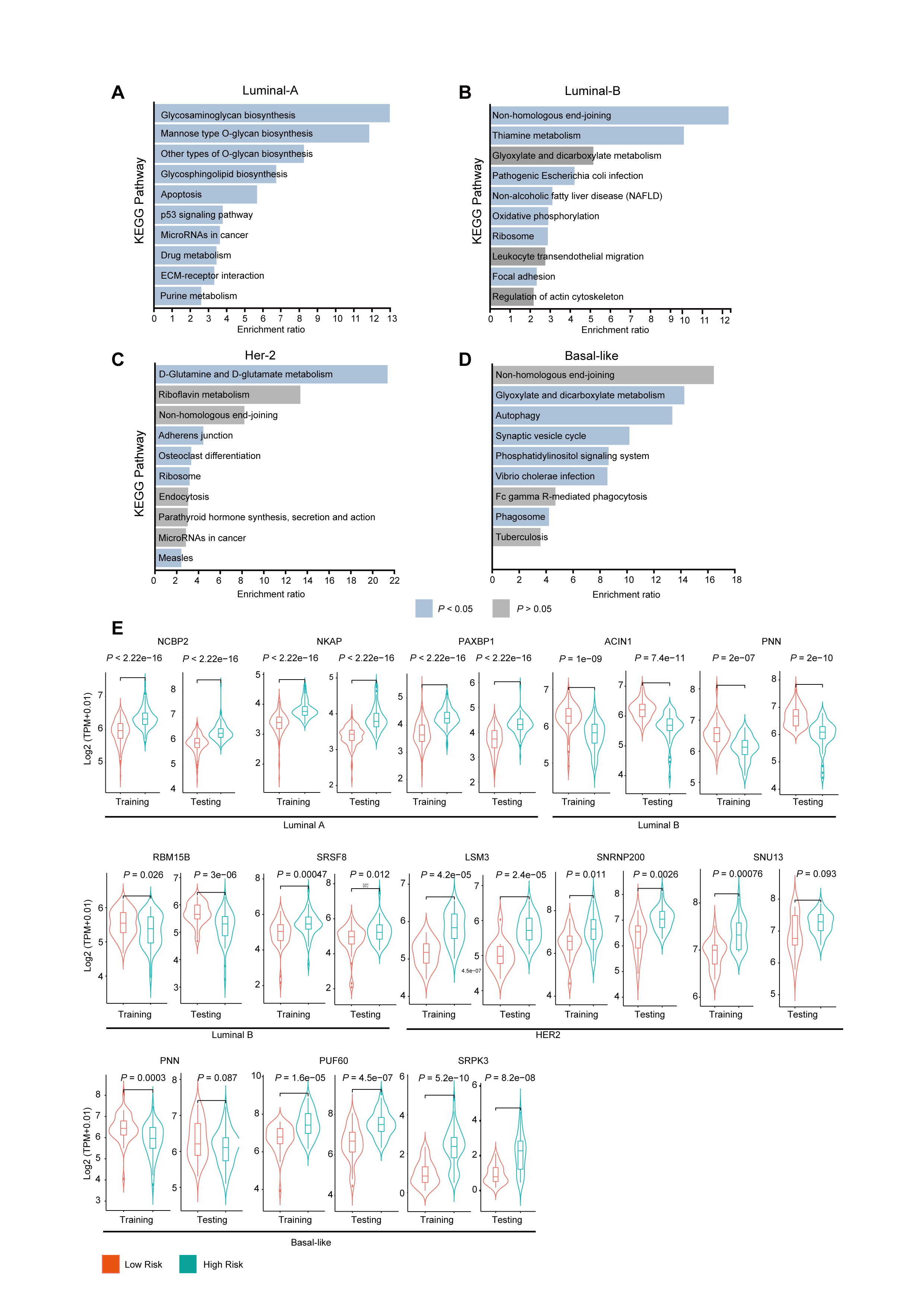

Supplement: Supplementary file 7 [file Image1.TIF]

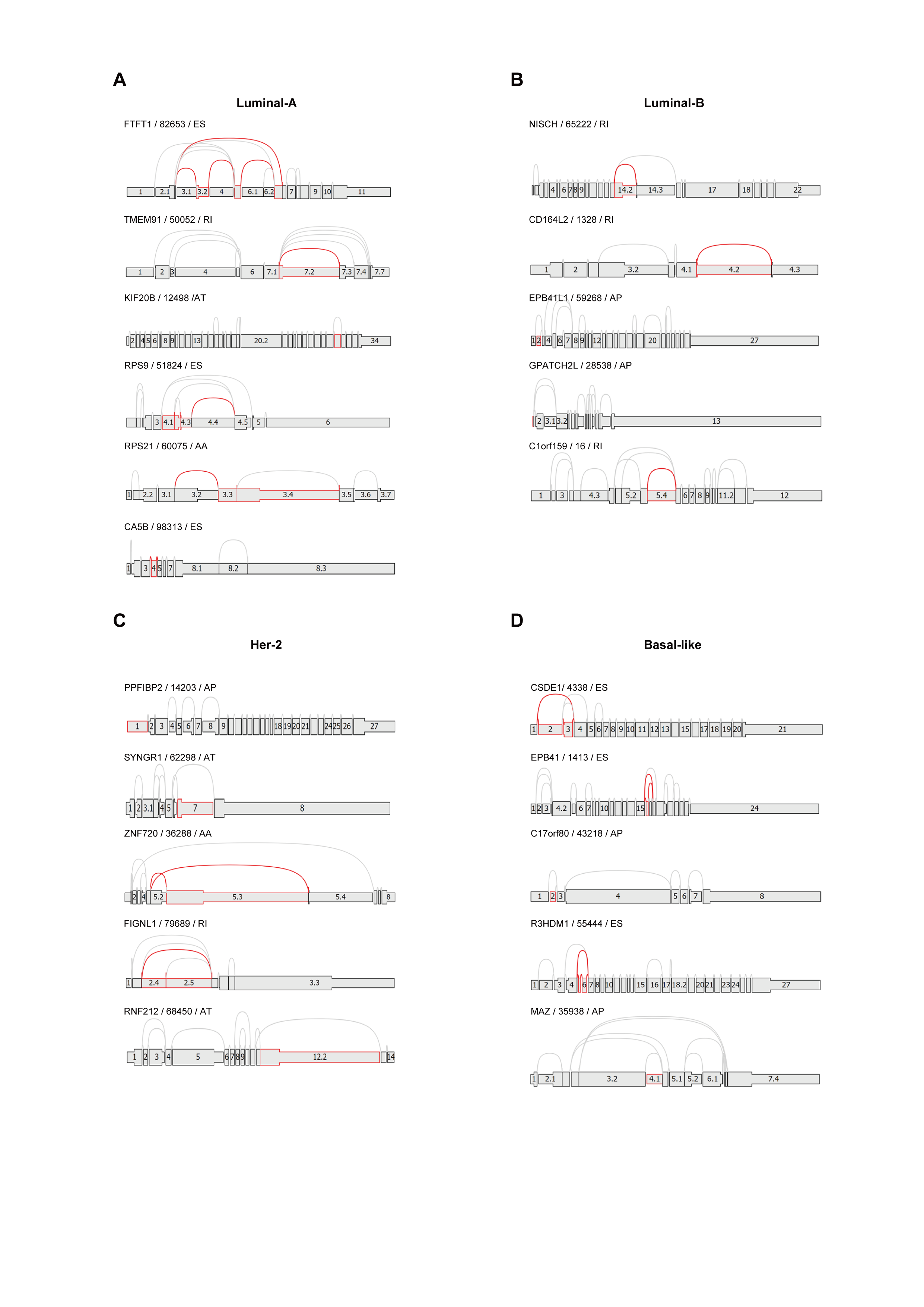

Supplement: Supplementary file 8 [file Image7.TIF]

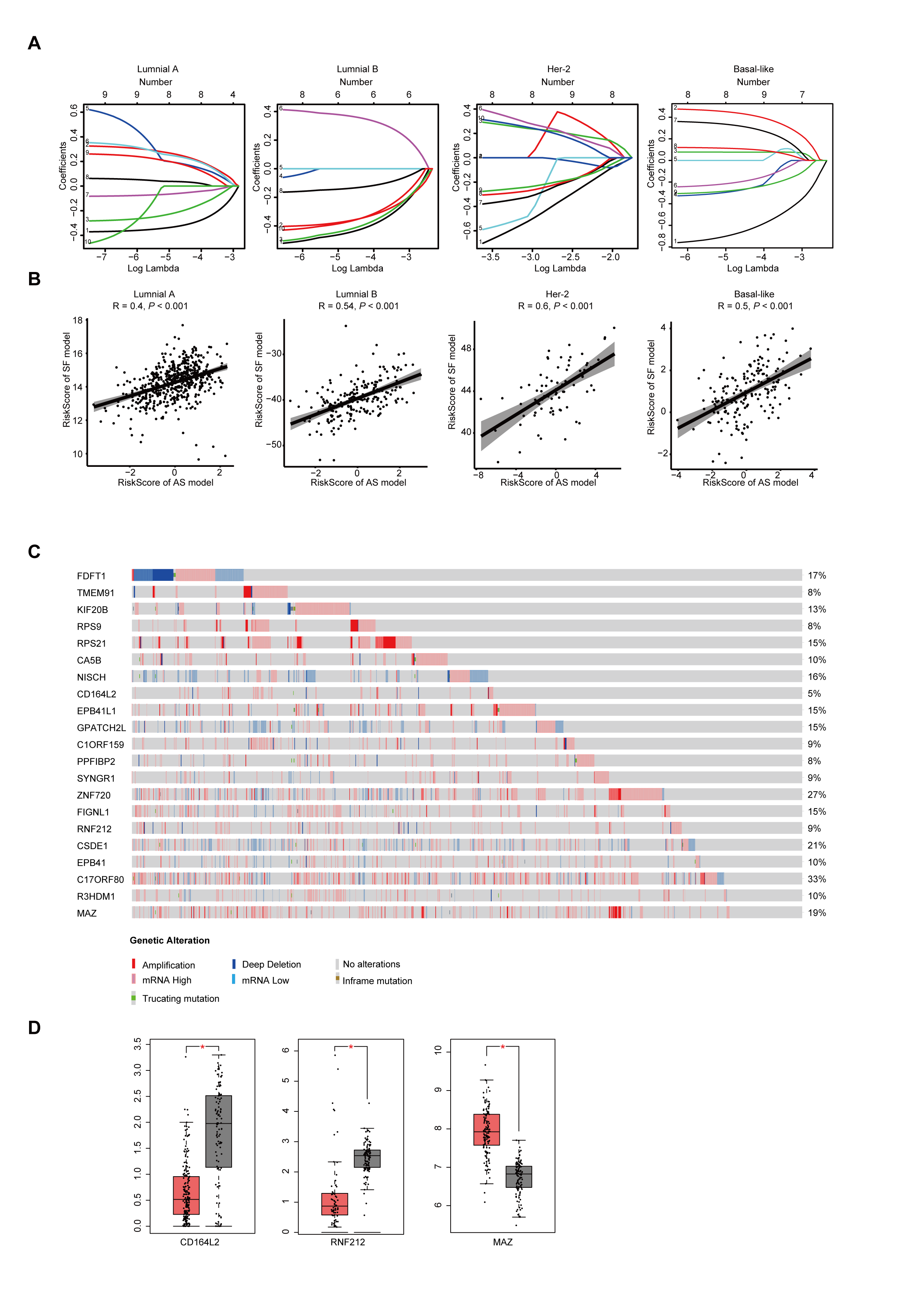

Supplement: Supplementary file 12 [file Image5.TIF]
