# Supplementary material for: Comprehensive Analysis of Splicing Factor and Alternative Splicing Event to Construct Subtype-Specific Prognosis-Predicting Models for Breast Cancer
Source: Front Genet. 2021 Sep 24;12:736423. doi: 10.3389/fgene.2021.736423 (PMC8497829; doi:10.3389/fgene.2021.736423)
Supplement: Supplementary file 15 [file DataSheet1.DOCX]

**Supplementary Figure Legends**

**Figure S1. KEGG enrichment analysis of survival-significant SFs in Luminal-A, Luminal-B, Her-2 and Basal-Like BRCA.**

**(A-D)** KEGG enrichment analysis of survival-significant SFs in Luminal-A **(A),** Luminal **(B)**, Her-2 **(C)** and Basal-like **(D)** BRCA. **(E)** Violin plots show the expression levels of genes in the risk models. The data shows the gene expression distribution in the high- and low-risk group (Wilcox-test).

**Figure S2. Kaplan-Meier analyses of SFs in SF-risk-models.**

**(A)** The lambda value selection for the Lasso regression. **(B-E)** Kaplan-Meier analysis of SFs in Luminal-A **(B)**, Luminal **(C)**, Her-2 **(D)** and Basal-like **(E)** BRCA.

**Figure S3. SFs in Luminal-A and Luminal-B risk-model are associated with prognosis in large cohorts of samples**

1. Kaplan-Meier analysis of SFs in Luminal-A using KM-plotter. **(B)** Kaplan-Meier

analysis of SFs in Luminal-B using KM-plotter.

**Figure S4. The mRNA expression of SF-risk-model related SFs in tumor and normal tissues of** **Luminal, Her-2, and Basal-like BRCA.**

**(A-D)** Boxplots showing the mRNA expression of related-SFs in Luminal-A **(A)**, Luminal **(B)**, Her-2 **(C)** and Basal-like **(D)** BRCA.

**Figure S5. Additional analysis of AS-risk-models.**

**(A)** LASSO regression in the construction of AS-riks-models. **(B)** Correlation of RiskScores predicted by SF-risk-models and AS-risk-models in Luminal, Her-2, and Basal-like BRCA. **(C)** Oncoprint analysis shows the genetic alterations of genes involved in the AS-risk-models. **(D)** Boxplots show the differentially expressed genes between tumor and normal tissue in the AS-risk-models.

**Figure S6. Schematic diagrams of the identified AS events.**

**(A)** Barplot shows the count of each alternative splicing category in the screened survival-related AS-events. **(B)** Barplot shows the percentage of each alternative splicing category in the screened survival-related AS-events.

**Figure S7. Schematic diagrams of the identified AS events.**

**(A-D)** Schematic diagrams showing the splicing pattern for the AS events identified in Luminal-A **(A)**, Luminal-B **(B)**, Her-2 **(C)** and Basal-like **(D)** AS-risk-models. All the splicing-pattern diagrams were obtained form TCGA SplicingSeq database (https://bioinformatics.mdanderson.org/TCGASpliceSeq/index.jsp).
